# Present College Session

**Published:** 1855-01

**Authors:** 


### PRESENT COLLEGE SESSION.

During the last session the very large increase of the class over any one session of former years was a subject of general remark, as well as congratulation, by the friends of the institution.

The present, however, far exceeds even the last session in the number of pupils, there being now nearly sixty, and eight or ten yet to come.

The public and the profession will require no better or surer evidence of the efficiency or the prudent management of the institution under the present Faculty, nor will either require a stronger guaranty of its future success, or its still more extended usefulness.

Two years have elapsed since it was thoroughly re-organized, when it became more decidedly a State institution, or in other words, it was so modified as to adapt it to its wants. The Faculty found it largely in debt, although the State had made a liberal appropriation for its benefit, which was entirely consumed. The manner of its disbursement, and the direction which it had taken, will be made known to the legislature; who will, doubtless, have the right to enquire into it, and it will be the duty of the Dean to report the particulars.

But although the burthen at first seemed almost insurmountable, yet the public good demanded that the effort should be made.—The work of regeneration was commenced under the most disheartening circumstances, and yet, after much toil, labor and anxiety, the Faculty have struggled through the difficulty, and have triumphantly placed the institution upon the high-way of success, beyond the expectation of its most ardent friends, and upon a firm and reliable basis; although menaced and opposed by low means and under-current measures, indicative of the minds and morals of its few enemies.

The first step in reorganization was the payment of debts which should have been met, as designed, by the fund appropriated. To do this, then, private and individual credit, and the private resources of individual members of the Faculty, were pledged and given. Some of the more pressing debts were wiped out and removed; others, through the magnanimity and generosity of the creditors, were postponed. Meanwhile, debts, of which the Faculty knew nothing previously, were most unexpectedly brought forward for payment. Although chagrined with the evidences of the previous recklessness and bad management, they persevered and met these also. At this time we can say that almost every claim against the College has been met, and our financial diifficulties removed. Such in part (and it constitutes a very small part) have been the difficulties under which the Faculty have labored, but as a reward for their perseverance they now see the school in the most prosperous condition; an honor to the State, the pride of the profession, and at the same time an object of the most tender solicitude on the part of the Faculty.

The same proportionate increase in the last two years would give the school one hundred pupils next session, and we do not entertain a doubt but that the number will be nearly up to these figures.

Our aim is to elevate the standard of the profession by sending forth from our halls men fully competent for the discharge of responsible duties of the profession. This is the great object, for we well know that the position and future welfare of the institution will depend upon a strict adherence to these principles. We will not suffer ourselves to be beguiled from our fixed and settled rule by any consideration of favoritism or any other influence, well knowing that while we pursue this course, we are substantially contributing to the best interests of community to an extent far beyond almost any other consideration. There is no one thing, perhaps, which will contribute more to the comfortable security of the citizens of a community, than the fact of the presence among them of enlightened and scientific medical men.

The profession, too, looks upon us with interest and indulge in ardent hopes for our success. We have the most abundant evidence of this. They see that within the limits of their own State, and at a trifling expense, they have abundant opportunities of prosecuting their enquiries into medical science, and of completing their studies.

Many have availed, and others are now availing, themselves of these benefits and privileges, heretofore so much desired.

To these and to all, we give the most solemn assurances, not by words only, but by our acts and efforts, of an unfailing devotion to our duty, embracing every requirement in our responsible position.

